# Supplementary material for: An improved ant colony optimization algorithm based on context for tourism route planning
Source: PLoS One. 2021 Sep 16;16(9):e0257317. doi: 10.1371/journal.pone.0257317 (PMC8445481; doi:10.1371/journal.pone.0257317)
Supplement: Attachment — Submitted filename: Reviewer Decision.docx [file pone.0257317.s001.docx]

PLOS ONE

PLOS ONE

Date: August 03,2021

Dear Editor-in-chief,

**Due to the subsequent paragraphs, I suggest the revision at this point.**

This paper deal with the new modification in ACO algorithm to solve a basic tourism route planning. The authors claimed that this new approach has enhanced the process of ACO to solve the problem. However, still some works must be done for better illustration of the whole manuscript. Please address the following comments and concerns:

1- Abstract is clearly outlined but the contribution of the study is not distinctively spelled out. Please consider. In addition, the structure of the abstract itself is problematic. The authors must start from more general viewpoint. Moreover, it is suggested to show the actual mathematical results by the means of improvement.
2- The introduction section is in fact “Introduction and Literature Review” section. While this is significant but the author mainly focused on the background rather than discussing their approach which must be think of. In addition, the utilized literatures are short and I cannot see the research gap at the end of this section. In addition, this section must end by a short complete overview of the following sections.
3- Some discussion in the literature review should be made, about the supply chain and supply chain management, consider the following works: Determination of the optimal sales level of perishable goods in a two-echelon supply chain network; Innovative approaches to design and address green supply chain network with simultaneous pick-up and split delivery; Determination of the economical policy of a three-echelon inventory system with (R, Q) ordering policy and information sharing; Deriving the cost function for a class of three-echelon inventory system with N-retailers and one-for-one ordering policy: An innovative waste management system in a smart city under stochastic optimization using vehicle routing problem; Utilizing IoT to design a relief supply chain network for the SARS-COV-2 pandemic.
4- The model assumption and model formulation are attractive. However, it would be more informative if the author could provide a diagram or flow chart to show the process of model development including model assumptions and model formulation. The Information is highly needed particularly for lay persons to understand the entire idea and objective of the paper.
5- While providing and developing different strategies, it would be more interesting to delve into the benefits and drawbacks of each strategy. It could be a real guide for various reader of this journal. In the case of proposed approach, it is suggested.
6- The utilized diagrams must be replaced with more quality pictures. Please consider.
7- Kindly provide the limitations of the adopted methods.
8- Conclusions should be rewritten to make it more constructive and conclusive, and should also explain the position of this paper in the existing research.
