# Supplementary material for: An improved ant colony optimization algorithm based on context for tourism route planning
Source: PLoS One. 2021 Sep 16;16(9):e0257317. doi: 10.1371/journal.pone.0257317 (PMC8445481; doi:10.1371/journal.pone.0257317)
Supplement: Attachment — Submitted filename: Response to Reviewers.docx [file pone.0257317.s002.docx]

## Response to Reviewers

Manuscript Number: PONE-D-21-21787

Date: August 25, 2021

Dear editor and dear reviewers:

Thank you for giving us the opportunity to submit a revised draft of the manuscript “A tourism route planning algorithm based on context feedback mechanism” for publication in the PLOS ONE. We appreciate the time and effort that the reviewers and editors dedicated to providing feedback on our manuscript and grateful for the insightful comments on and valuable improvements to our paper.

We have incorporated most of the suggestions made by the reviewers. Those changes are highlighted within the manuscript. Please see below, in blue, for a point-by-point response to the reviewers’comments and concerns. All page numbers refer to the revised manuscript file with tracked changes.

Reviewers’ Comments to the Authors:

Reviewer 1:

1. The title of the paper is not suitable. I think you must use your novelties and the overall image of your paper.

**Author response: As suggested by the reviewer, according to the novelties of our improved algorithm: change the ant colony pheromone distribution mechanism based on contextual factors, so as to dynamically adjust the tourism route planning to maximize the benefits of tourists. The new title is “An improved ant colony optimization algorithm based on context for tourism route planning”.**

2. I encourage you to add more detail about your core contributions in the abstract.

**Author response: We agree with the reviewer’s assessment. Accordingly, we reconstructed the abstract and emphasized the optimization ideas of the ACOCA algorithm. In addition, through experimental results and comparative analysis with other algorithms, the advanced nature of the improved algorithm is verified. See the ‘Abstract’ section on page 1 of the revised manuscript.**

3. Literature review is very short and old! You have not covered the knowledge edge! Please clarify the contribution of the paper according to the research gap.

**Author response: We have added the suggested content to the manuscript on the “Introduction” section, some recent ACO algorithm literatures review is added in page2 (Line 42 - Line 63). We have pointed out the research gap on paragraph 3, page3 (Line 73-Line 85) and paragraph 1, page 5 (Line 160-Line164). See more on page 4 and page 6 of the revised manuscript.**

4. Many recent papers in the area can be added to the literature review. I do not propose you a especial reference due to reviewing ethical issues.

**Author response: As suggested by the reviewer, we have added some recent papers in the area, such as supply chain management on the “ACO algorithm” section. See on paragraph 3, page 4 (Line 152 - Line 159).**

5. Check the English presentation of this paper to remove the typos mistakes.

**Author response: Thank you for pointing this out. The revised text reads as follow on:**

(1) **We have modified the tense of some sentences, for example, the first sentence on paragraph 2 page 2 (Line 10 - Line 11).**
(2) **We have corrected the expression of some terms, for example, change “ant colony algorithm” to “ant colony optimization algorithm” (Line 124, Line 131).**
(3) **We have removed some typos mistakes.**
6. Findings, limitations, and recommendations of this paper can be discussed more in the conclusion section.

**Author response: As suggested by the reviewer, the last section “Conclusion” has been revised, firstly, the title of this section renamed “conclusion and future work”; secondly, on the first paragraph of this section, we discuss the findings, limitations of this paper; finally, we introduce the future work of this research field. See details on the page 12 (Line 368 - Line 388).**

7. Please draw and bring some better figures in terms of color and quality.

**Author response: Thank you for pointing this out. The reviewer is correct, and we have repainted the illustrations, especially the colors of figure 1, figure 3, figure 4, and figure 5 to improve the display quality of the images.**

Reviewer 2:

1. Abstract is clearly outlined but the contribution of the study is not distinctively spelled out. Please consider. In addition, the structure of the abstract itself is problematic. The authors must start from more general viewpoint. Moreover, it is suggested to show the actual mathematical results by the means of improvement.

**Author response: As the reviewer suggested, we have made substantial revisions in the abstract section. On the one hand, we pointed out the shortcomings of the ACO algorithm in solving the problem of tourism route planning, and focused on the innovations of our work, and improved the update strategy of ant colony pheromone through the contextual information feedback mechanism of the scenic spot. Thereby improving the dynamic adjustment ability of tourism route planning, and highlighting the improvement of the user's travel experience. On the other hand, through experiments and comparison with the baseline algorithm, it was verified that the ACOCA algorithm has improved in terms of route distance, time overhead, and comfort. See “Abstract” section on page 1 of the revised manuscript.**

2. The introduction section is in fact “Introduction and Literature Review” section. While this is significant but the author mainly focused on the background rather than discussing their approach which must be think of. In addition, the utilized literatures are short and I cannot see the research gap at the end of this section. In addition, this section must end by a short complete overview of the following sections.

**Author response: According this suggestion, we have made some revisions as following:**

(1) **We have added one paragraph at the end of the “Introduction” section. In this paragraph, we introduce the structure of this paper. See more on paragraph 2, page 2 (Line 33- Line 54).**
(2) **Some recent ACO algorithm literature review is added on paragraph 3 page 4 and point out the research gap on the “Introduction” section on paragraph 3, page 3 (Line 73-Line 85).**
3. Some discussion in the literature review should be made, about the supply chain and supply chain management, consider the following works: Determination of the optimal sales level of perishable goods in a two-echelon supply chain network; Innovative approaches to design and address green supply chain network with simultaneous pick-up and split delivery; Determination of the economical policy of a three-echelon inventory system with (R, Q) ordering policy and information sharing; Deriving the cost function for a class of three-echelon inventory system with N-retailers and one-for-one ordering policy: An innovative waste management system in a smart city under stochastic optimization using vehicle routing problem; Utilizing IoT to design a relief supply chain network for the SARS-COV-2 pandemic.

**Author response: We have added the suggested content to the manuscript on the “ACO algorithm” section, on paragraph 3, page 4 (Line 152- Line 159). We have added some literature review in this paragraph, such as supply chain management, vehicle route planning, etc.**

4. The model assumption and model formulation are attractive. However, it would be more informative if the author could provide a diagram or flow chart to show the process of model development including model assumptions and model formulation. The Information is highly needed particularly for lay persons to understand the entire idea and objective of the paper.

**Author response: It’s a good idea for model formulation, we have given the cost optimization formula and the definition of related constraints for tourism route planning, See Eq. 13 to Eq. 19 for details in the revised manuscript.**

5. While providing and developing different strategies, it would be more interesting to delve into the benefits and drawbacks of each strategy. It could be a real guide for various reader of this journal. In the case of proposed approach, it is suggested.

**Author response: As the reviewer’s suggestion, we elaborated the ACOCA algorithm design strategy: on the one hand, the pheromone update strategy of the ant colony is dynamically adjusted according to the contextual information of the scenic spot, so that tourists tend to be suitable for visiting routes; on the other hand, the support degree of sub-path is set to prevent the algorithm from falling into the local optimal. See more on “Proposed method” section, (Line 204-Line 208).**

6. The utilized diagrams must be replaced with more quality pictures. Please consider.

**Author response: Thank you for pointing this out. The reviewer is correct, and we have repainted the illustrations, especially the colors of figure 1, figure 3, figure 4, and figure 5 to improve the display quality of the images.**

7. Kindly provide the limitations of the adopted methods.

**Author response: We agree that this is a potential limitation of the study. We have added this as a limitation on the end of “Conclusion and future work” section.**

8. Conclusions should be rewritten to make it more constructive and conclusive, and should also explain the position of this paper in the existing research.

**Author response: We refactored the “conclusion” section, and made the following changes:**

**(1) Summarized the main innovations of the improved ACOCA algorithm in this paper;**

**(2) Summarized the experiments on two different datasets, compared with the performance of the baseline method, and verified the advantages of the proposed method;**

**(3) Finally, the shortcomings of the ACOCA algorithm are also introduced, and the future research is also prospected.**

Academic Editor:

Journal Requirements:

1. When submitting your revision, we need you to address these additional requirements.

**Author response: Yes, we have provided standard manuscript and illustrations in this revised version, include a rebuttal letter labeled ‘Response to Reviews’, a marked-up copy of our manuscript, an unmarked version of revised paper without tracked changes and figures.**

2. We note that Figure 2 in your submission contain [map/satellite] images which may be copyrighted. All PLOS content is published under the Creative Commons Attribution License (CC BY 4.0), which means that the manuscript, images, and Supporting Information files will be freely available online, and any third party is permitted to access, download, copy, distribute, and use these materials in any way, even commercially, with proper attribution. For these reasons, we cannot publish previously copyrighted maps or satellite images created using proprietary data, such as Google software (Google Maps, Street View, and Earth).

**Author response: Yes, Figure 2 includes a map. Figure 2 is a visualization image generated by Python's folium package, which folium builds on the data wrangling strengths of the Python ecosystem and the mapping strengths of the Leaflet.js library. Manipulate our data in Python, then visualize it in a Leaflet map via folium. Leaflet is an open-source JavaScript library for mobile-friendly interactive maps, the map data comes from** OpenStreetMap[^®^](https://www.openstreetmap.org/copyright/en#trademarks)**. There is a copyright and license introduction about OpenStreetMap:**

OpenStreetMap[^®^](https://www.openstreetmap.org/copyright/en#trademarks) is *open data*, licensed under the [Open Data Commons Open Database License](https://opendatacommons.org/licenses/odbl/) (ODbL) by the [OpenStreetMap Foundation](https://osmfoundation.org/) (OSMF).

You are free to copy, distribute, transmit and adapt our data, as long as you credit OpenStreetMap and its contributors. If you alter or build upon our data, you may distribute the result only under the same licence. The full [legal code](https://opendatacommons.org/licenses/odbl/1.0/) explains your rights and responsibilities.

Our documentation is licensed under the [Creative Commons Attribution-ShareAlike 2.0](https://creativecommons.org/licenses/by-sa/2.0/) license (CC BY-SA 2.0).

**(source: https://www.openstreetmap.org/copyright/en)**

**Therefore, the map in Figure 2 does not involve copyright issues. For more information please visit the folium website:** [**https://pypi.org/project/folium/**](https://pypi.org/project/folium/)**, openstreetmap website:** **https://www.openstreetmap.org/.**

**However, in order to ensure compliance with copyright requirements, we have redrawn Figure 2, using a plane coordinate system to indicate the location of the scenic spot.**
